# Total Oxidative status of Mouse Vitrified Pre-Antral Follicles with Pre-Treatment of Alpha Lipoic Acid

**DOI:** 10.6091/ibj.1258.2014

**Published:** 2014-07

**Authors:** Sahar Hatami, Saeed Zavareh, Mojdeh Salehnia, Taghi Lashkarbolouki, Mohammad Taghi Ghorbanian, Isaac Karimi

**Affiliations:** 1 *School of Biology, Damghan University, Damghan, Iran; *; 2 *Institute of Biological Sciences, Damghan University, Damghan, Iran;*; 3 *College of Medical Sciences, Tarbiat Modares University, Tehran, Iran; *; 4 *Laboratory of Molecular and Cellular Biology, Department of Basic Veterinary Sciences, School of Veterinary Medicine, Razi University, Kermanshah, Iran*

**Keywords:** Vitrification, Pre-antral follicle, Alpha lipoic acid (ALA), Reactive oxygen species (ROS), Total antioxidant capacity (TAC)

## Abstract

**Background: **Cryopreservation of pre-antral follicles is a hopeful technique to preserve female fertility. The aim of the present study was to evaluate reactive oxygen species (ROS) and total antioxidant capacity (TAC) levels of mouse vitrified pre-antral follicles in the presence of alpha lipoic acid (ALA). **Methods: **Isolated pre-antral follicles (140–150 µm in diameter) were divided into vitrified–warmed and fresh groups. Each group was subjected to *in vitro* maturation with or without ALA for 12 days, followed by adding human chronic gonadotropin to induce ovulation. *In vitro* fertilization was performed to evaluate their developmental competence. In parallel, the amount of ROS and TAC were assessed after 0, 24, 48, 72, and 96 h of culture by 2',7'-dichlorofluorescin assay and ferric reducing/antioxidant power assay, respectively. **Results: **The respective rates of survival, antrum formation, and metaphase II oocytes were significantly higher in ALA-supplemented groups compared to the groups not treated with ALA. TAC and ROS levels were significantly decreased and increased, respectively during the culture period up to 96 h in the absence of ALA in both vitrified and non-vitrified samples. However, with pretreatment of ALA, TAC levels were increased significantly and remained constant up to 96 h in vitrified-warmed pre-antral follicles, while ROS levels completely returned to the level of starting point after 96 h of culture in the presence of ALA. **Conclusion: **Pretreatment of ALA positively influences development of pre-antral follicles in vitrified and non-vitrified samples through increasing follicular TAC level and decreasing ROS levels.

## INTRODUCTION

Despite much progress in cryopreservation of ovarian tissue and grafting procedures, some obstacles such as postgrafting ischemia [1] and risk of transmitting malignant cells present in cryopreserved tissue [2], which leads to yield unsatisfactory results. To avoid these problems, culture of isolated pre-antral follicles was proposed. This procedure would allow the evaluation of follicular quality after cryopreservation [3] and investigate metabolism of follicle during the culture period and somewhat preserve the female fertility. 

Oxidative metabolism is indispensable for energy production of follicle, which in turn results in generation of reactive oxygen species (ROS). Although a critical amount of ROS is essential for their physiological activities, excessive amount of them causes oxidative stress [4]. In addition, there is a relationship between oxidative stress and oocyte quality [5]. It is known that certain levels of ROS and total antioxidant capacity (TAC) improve developmental competence of cultured pre-antral follicles [6]. 

In the *in vivo *condition, generation of ROS is equilibrated by enzymatic and non-enzymatic antioxidant defense systems of cells [7, 8]. However, the lack of this physiological defense system in the *in vitro* condition with high pressure of oxygen can lead to oxidative stress [9]. Assessments of ROS and TAC during *in vitro* culture is important for evaluating the quality of cultured follicles [10].

Alpha lipoic acid (ALA) naturally presents in mitochondria as a cofactor for pyruvate dehydrogenase and α-ketoglutarate dehydrogenase [11, 12]. In addition, antioxidant properties of ALA in *in vitro *conditions have received attention [6, 11]. ALA can directly scavenge ROS and indirectly recycle other intracellular antioxidants [11, 12]. We have recently demonstrated that ALA improves the developmental competence of follicles through improving oxidative status of follicles during culture period [6].

On the other hand, it has been shown that cryodamage after ovarian cryopreservation results in oxidative stress [10, 13], which in turn attributed to change in the integrity of cell membrane and mitochondrial function [14]. To date, there is no study indicating oxidative status of mouse vitrified-warmed pre-antral follicles. Hence, the objective of the present study was to evaluate the effect of ALA on oxidative status of mouse vitrified-warmed pre-antral follicles during *in vitro* culture.

## MATERIALS AND METHODS

***Reagents.*** All reagents were obtained from Sigma-Aldrich (UK) unless otherwise stated.

***Animals and experimental design. ***Female NMRI mice were purchased from Pasteur Institute of Iran (Tehran). The animal protocol was approved by Animal Care and Use Committee of the Damghan University (Semnan Province, Iran). Animals were housed and bred under light and temperature controlled conditions (12-h light and 12-h darkness at 24°C) and provided with food and water *ad libitum*. For all experiments, 14-15-day-old mice were killed by cervical dislocation, and their ovaries were transferred to α-MEM (Gibco, UK), supplemented with 10% FBS (Gibco, UK), 2.2 g/L sodium bicarbonate, 25 mM HEPES, 100 IU/ml penicillin, and 75 µg/ml streptomycin. The ovaries were randomly divided into 2 groups: (1) those that not vitrified (control) and (2) those that their follicles were isolated and then vitrified. Isolated pre-antral follicles of each group were cultured in the presence or absence of 100 µM ALA. 

***Pre-antral follicle isolation. ***Pre-antral follicles were isolated mechanically by using a 29-gauge needle under a stereomicroscope (Nikon, Japan) at 10× magnification. Isolated follicles were selected according to the following criteria: rounded follicular structure with 140-150 µm diameters containing visible centrally located healthy oocyte surrounded with intact, several layers of granulosa cells, an intact basement membrane, at least one layer of theca cells without antral cavity. During the operating procedures, the culture medium was constantly kept at 37°C.

***Vitrification and warming of pre-antral follicles. ***The vitrification procedure was based on methods described previously [15] with some modifications. In brief, isolated pre-antral follicles were initially incubated at room temperature for 5 minutes in equilibrium solution containing 7.5% (v/v) ethylene glycol and 7.5% (v/v) DMSO in Dulbecco phosphate-buffered saline (DPBS) medium (Gibco, UK) with 20% FBS. Then, they were incubated in vitrification solution containing 15% ethylene glycol, 15% DMSO, and 0.5 M sucrose in DPBS + 20% FBS for 30 s. After dehydration, pre-antral follicles were immediately removed with a minimum volume of vitrification solution (<0.1µl). They were then individually placed on top of a polypropylene strip of cryotop carrier (Kitazato, Japan) and immediately immersed into liquid nitrogen. The thin strip of cryotop was covered with a cap and stored in a liquid nitrogen tank for at least one week. All equilibration and vitrification steps were carried out at room temperature. For warming, the cryotop cap was removed, while immersing in liquid nitrogen. The strip was directly submerged in DPBS medium containing 1 M sucrose. The pre-antral follicles were left in the warming solution (1 M sucrose in DPBS) for 90 s and transferred into droplets of DPBS medium containing 0.5 and 0.25 M sucrose at room temperature at an interval of 5 min. Next, pre-antral follicles were pipetted into a fresh α-MEM medium supplemented with 10% FBS at 37°C for another 10 min before placing in the culture medium.

***In vitro culture of isolated pre-antral follicles.*** *In vitro* culture of the isolated fresh and vitrified/warmed pre-antral follicles was performed according to our previous methodology [6]. Briefly, fresh and vitrified/warmed pre-antral follicles were cultured individually in 20-µl droplets of α-MEM (pH 7.2), supplemented with 0.23 mM sodium pyruvate, 5% FBS, 100 mIU/ml recombinant follicle stimulating hormone (Gonal-F, Switzerland), 10 mg/ml insulin, 5.5 mg/ml transferrin, 0.67 mg/ml insulin transferrin selenium (Gibco, UK), 20 ng/ml murine recombinant epidermal growth factor, 100 IU/ml penicillin, 75 µg/ml streptomycin, 2.2 g/L sodium bicarbonate, and 100 µM ALA. This process was performed according to experimental design under mineral oil in a humidified atmosphere of 5% CO_2_ in air at 37°C in Falcon Petri dishes (60 × 15 mm^2^) for up to 12 days. At 48-h intervals, 10 µl of culture medium from each drop was replenished by a fresh medium.

***Evaluation of morphological and developmental parameters of cultured pre-antral follicles. ***The survival rate of the pre-antral follicles was assessed microscopically based on the morphology of the pre-antral follicle every other day under an inverted microscope during culturing period. Then, they were compared at the end of the study as described previously [16]. Briefly, a follicle was considered normal when it possessed a centrally located spherical and also homogeneous oocyte surrounded by complete and compact layers of granulosa cells without noticeable damage to the basement membrane. The follicles with partially or completely naked oocytes or those with morphological signs of degeneration, such as darkness of oocytes and surrounding cumulus cells, or follicles with misshapen oocytes were graded as degenerated. Antral-like cavity formation was considered as a visible, lucent area in the granulosa cell mass around the oocyte. Follicle diameter was measured only in healthy follicles on days 2 and 4 of culturing period. Mean follicle diameter was assessed by measuring and averaging two perpendicular cross-sectional diameters of each follicle with pre-calibrated ocular micrometer under an inverted microscope (Nikon, Tokyo, Japan, 100× magnification [6]). 

***In vitro ovulation induction. ***In the 10^th^ day of culture, ovulation and final oocyte maturation were induced by substitution of culture media with fresh maturation medium supplemented with 1.5 IU/ml human chronic gonadotropin (hCG; IBSA, Switzer-land). Then, 24 h after hCG addition in all groups, released oocytes were classified as germinal vesicle, germinal vesicle breakdown when the germinal vesicle was absent, and metaphase II (MII) oocytes when the first polar body was extruded. 

***In vitro fertilization. ***The fertile NMRI male mice were sacrificed by cervical dislocation and their cauda epididymis dissected and placed into a 500-µl drop of modified Tyrode's medium supplemented with 5% BSA under mineral oil. The caudae epididymis were ripped with the aid of a 28-gauge needle, and the sperms were squeezed out gently by using forceps and incubated in 5% CO_2_ at 37°C for 90 min to allow the spermatozoa to swim out and capacitate. Capacitated sperm suspension was added to MII oocyte to give the final motile sperm concentration of 1-2 × 10^6^ /ml. MII oocytes and sperms were co-incubated in a modified KSOM-AA medium [17] in 5% CO_2_ at 37°C for 6 h. Oocytes presenting two pronuclei were considered normally fertilized. Fertilized oocytes were washed and gently pipetted to remove spermatozoa and attached to cumulus cells. Zygotes were then transferred to drops of fresh KSOM-AA medium and cultured in 5% CO_2_ with maximum humidity at 37°C until day 5. Day of fertilization was considered as day 0. Embryos were observed on the heated stage (37°C) with an inverted microscope at 200× magnification (Nikon, TE2000-U with Hoffman modulation contrast). The numbers of zygotes that reached the two cells, morula and blastocytes were counted.

***Biochemical assays***

***Assessment of reactive oxygen species. ***The intracellular ROS production of fresh and vitrified-warmed pre-antral follicles was measured as described previously [6, 10]. Briefly, 10 follicles were used for each measurement at different times of culturing period (0, 24, 48, 72, and 96 h), in each study group. After washing with PBS, follicles were incubated in 40 mmol/l Tris-HCl buffer (pH 7.0) containing 5 µM of 2',7'-dichlorodihydrofluorescin diacetate (Merck, Germany) at 37°C for 30 min. Follicles were then washed again with PBS and then transferred to 100 µl Tris-HCl buffer (40 mmol/l, pH 7.0) and sonicated at 50 W for 1 min. Solution was centrifuged at 10,000×*g* at 4°C for 20 min, and fluorescent intensity of supernatant was monitored by using a spectro-fluorometer at 525 nm emission and 488 nm excitation. Corrections for autofluorescence were made by including parallel blanks in each experiment. Values for ROS levels were expressed as µM H_2_O_2 _produced. All experiments were repeated at least five times.

***Total antioxidant capacity assay.*** Assessment of TAC in cultured pre-antral follicles was performed according to ferric reducing/antioxidant power method [6, 10]. Briefly, in order to prepare cellular supernatant, 10 follicles were collected at different time periods (0, 24, 48, 72, and 96 h) and homogenized in 100 ml Tris–HCl buffer (40 mmol/l, pH 7.0). They were then sonicated at 50 W for 1 min and centrifuged at 10,000×*g* at 4°C for 20 min. Cellular supernatant (100 µl) was added to 1 ml fresh ferric reducing/antioxidant power reagent (tripyridyltriazine; Merck) in a cuvette and incubated at 37°C for 10 min. Absorbance of blue-colored reagent containing 300 mL ferric reducing/ antioxidant power reagent plus 10 mL distilled water at 593 nm every 20 s for 10 min. Standard solutions of Fe^2+^ in the range of 100–1000 mM were prepared from ferrous sulfate in distilled water. All experiments were repeated at least five times.

***Statistical analysis. ***Each experiment was carried out at least in four replicates. The data were presented as the mean standard deviation (SD). Statistical analysis was performed using SPSS software package ver. 16 (SPSS Inc., Chicago, IL, USA). All proportional data were analyzed after arcsine of sqrt transformation. Differences in groups were evaluated by one-way analysis of variance (ANOVA), and Tukey's HSD was used as *post hoc* test. Differences were considered significant at a level of *P*<0.05. 

**Fig. 1:**
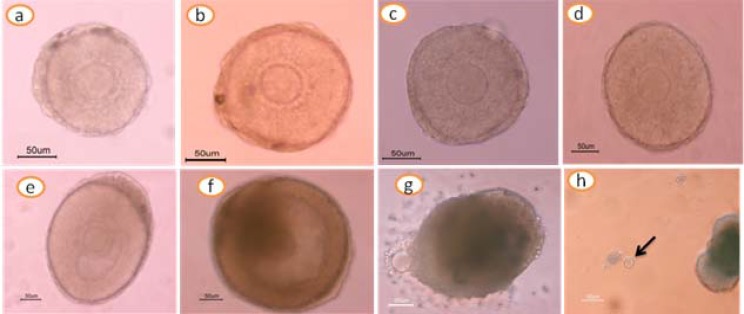
Cultured pre-antral follicle: initial day **(a)**, day 2 **(b)**, day 4 **(c)**, day 6 **(d)**, day 8 **(e)**, day 10 **(f)** day 12 **(g)**, and ovulated MII oocyte after hCG stimulus **(h)**.

## RESULTS

Cultured follicles were evaluated morphologically every other day. After the fourth day of culture, granulosa cells were proliferated and grew through the basal membrane to form the irregular and diffuse appearance that was impossible to measure follicle diameter (Fig. 1). The mean follicle diameter at the beginning of the culture (147.9 ± 5.9 µm) was not significantly different among all groups (*P*>0.05). During the culture period, the follicle diameter was increased progressively (Table 1). 

By day 12 of cultivation period, there were significantly differences in both vitrified and non-vitrified samples with pretreatment of ALA compared with ALA-free conditions groups (Tables 1 and 2, *P*<0.05) with regard to the follicle diameter and rates of survival, antrum formation, and oocyte maturation. There were no significant differences between diameters of vitrified-warmed follicles with ALA pretreatment and fresh follicles without ALA pretreatment on days 2 and 4 (Table 1). The rates of survival (68.3%) and antrum formation (62.8%) in the vitrified-warmed pre-antral follicles were significantly lower than those in fresh pre-antral follicles (85% and 76.7% respectively). The survivance and antrum formation of ALA-treated vitrified-warmed pre-antral follicles and fresh pre-antral follicles were also comparable (*P*>0.05) (Table 2).

A higher percentage of extruded MII oocytes (53.9%) were achieved from the cultured pre-antral follicles after addition of the hCG in fresh pre-antral follicles with ALA pretreatment (*P*<0.05). The rate of MII oocytes was similar between cultured fresh pre-antral follicles and vitrified-warmed pre-antral follicles with pretreatment of ALA (Table 2).

The rates of fertilization and blastocyte formation (Table 3) were statistically lower in vitrified-warmed pre-antral follicles compared to control group (*P*<0.05). Also, significant higher rates of fertilization and blastocyte formation were achieved in respective ALA-supplemented groups both in vitrified and non-vitrified groups (*P*<0.05). There were no significant differences in the rates of fertilization and blastocyst development between ALA pretreated vitrified-warmed pre-antral follicles and control groups.

**Table 1:** Diameter of cultured vitrified-warmed and fresh pre-antral follicles supplemented with or without alpha lipoic acid (ALA

**Groups**	**ALA**	**NO. of** **follicle** **s**	**Follicle diameter (** **µm ± SD)**		
			**0** ^th^ ** day**	**2** ^th^ ** day**	**4** ^th^ ** day**
Fresh follicles	-	240	148.2 ± 5.3	189.6 ± 11.4^a^	290.5 ± 26.3^a^
	+	180	147.5 ± 6.6	223.9 ±17.1^b^	323.3 ± 11.6^b^
					
Vitrified pre-antral follicles	-	180	148.3 ± 5.0	175.3 ± 7.2^c^	264.2 ± 6.7^c^
	_+_	180	148.6 ± 5.7	180.5 ± 7.2^a^	272.1± 18.4^a^

In all cases at least 4 experimental replicates were performed. Different superscripts in the same column reflect significant difference (*P*<0.05)

**Table 2:** Maturation rates of cultured vitrified-warmed and fresh pre-antral follicles supplemented with or without ALA

**Groups**	**ALA**	**NO.**	**Survived** **n** **(% ± SD)**	**Degenerated** **n** **(% ± SD)**	**Antrum formation** **n (% ** **± SD)**	**Maturation stages of oocyte**		
						**GV** **n (% ** **± SD)**	**MI** **n (% ** **± SD)**	**MII** **n (% ** **± SD)**
Fresh follicles	**-**	240	204^a^ (85.0 ± 1.9)	36^a^ (15.0 ± 1.9)	184^a^ (76.7 ± 5.6)	30 (12.5 ± 3.5)	51 (21.2 ± 3.7)	103^a^ (42.9 ± 2.8)
	**+**	180	169^b^ (94.3 ± 1.2)	11^b^ (5.7 ± 1.2)	166^b^ (92.3 ± 4.0)	16 (9.5 ± 2.4)	34 (18.9 ± 5.1)	97^b^ (53.9 ± 4.2)
								
Vitrified pre-Antral follicle	**-**	180	123^c^ (68.3 ± 6.0)	57^c^ (31.7 ± 6.0)	113^c^ (62.8 ± 4.2)	21 (11.7 ± 1.7)	20 (11.1 ± 2.5)	54^c^ (28.3 ± 1.7)
	**+**	180	150^a^ (83.3 ± 3.3)	30^a^ (16.7 ± 3.3)	134^a^ (74.4 ± 4.2)	20 (11.1 ± 2.5)	21 (11.7 ± 1.7)	66^a^ (36.7 ± 3.3)

In all cases, at least 4 experimental replicates were performed. Different superscripts in the same column reflect different levels of significant difference (*P*<0.05). ALA, alpha lipoic acid; GV, germinal vesicle stage oocyte; MI: metaphase I oocyte; MII: metaphase II oocyte

**Fig. 2:**
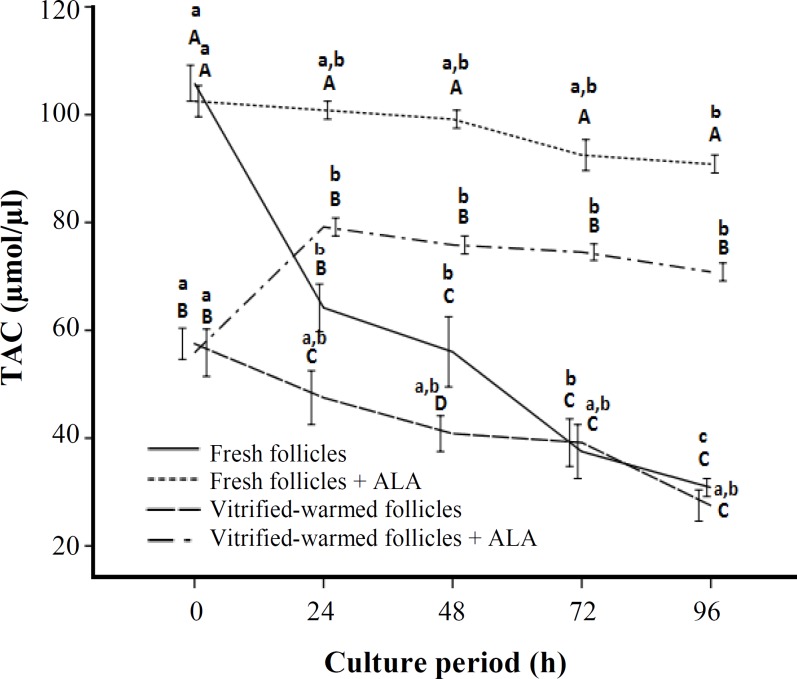
Total antioxidant capacity (TAC) levels in cultured vitrified-warmed and fresh pre-antral follicles with or without pretreatment of alpha lipoic acid (ALA). In all cases 4 experimental replicates were performed. Different superscripts (A, B, C) reflect different levels of significant differences at same times of culture among the different groups (*P*<0.05). Different superscripts (a, b, c) reflect different levels of significant differences at different times of cultivation period within the same group (*P*<0.05).

***Total antioxidant capacity*** ***levels of cultured isolated pre-antral follicles. ***TAC levels in non-vitrified and vitrified pre-antral follicles after 0, 24, 48, 72, and 96 h of culture with or without pretreatment of ALA are shown in Figure 2. At the beginning of culture, TAC level was significantly higher in the fresh pre-antral follicles (105.8 µmol/µl) compared to vitrified-warmed pre-antral follicles (57.5 µM/µl, *P*<0.05). There were significant differences between TAC levels in fresh pre-antral follicles and vitrified-warmed pre-antral follicles at 24, 48, 72, and 96 h of culture (*P*<0.05). Up to 96 h of culture period, TAC levels were significantly decreased in the fresh and vitrified pre-antral follicles without pretreatment of ALA (*P*<0.05). However, in the presence of ALA, TAC levels were remained constant without significant difference in the fresh pre-antral follicles and in vitrified-warmed pre-antral follicles. These levels were increased significantly after 24 h of culture and remained constant without significant difference up to 96 h later. 

**Fig. 3:**
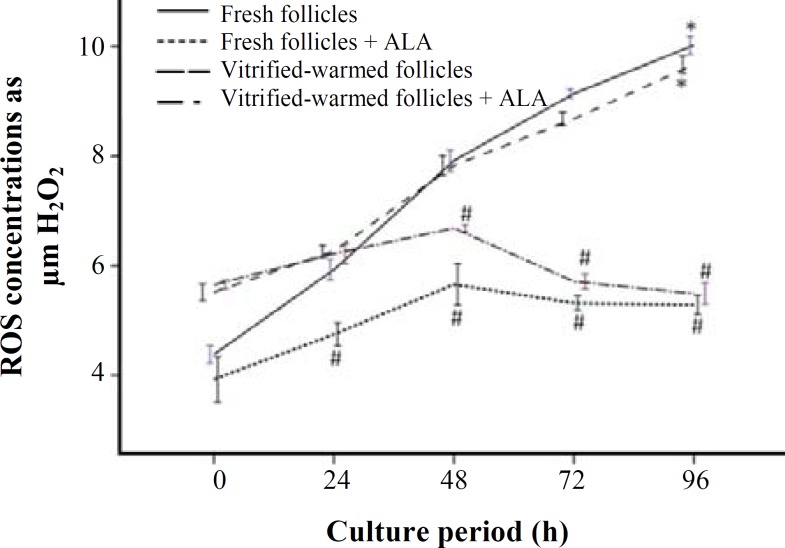
Reactive oxygen species (ROS) concentrations in cultured vitrified-warmed and fresh pre-antral follicles with or without pretreatment of alpha lipoic acid (ALA). In all cases 4 experimental replicates were performed. * indicates significant difference with initial time in the same group (*P*<0.05); **# **indicates significant difference at same times of cultivation period between respective groups with or without pretreatment of ALA (*P*<0.05). ROS, reactive oxygen species; ALA, Alpha lipoic acid

***Reactive oxygen species*** ***production in cultured pre-antral follicles. ***ROS levels in vitrified and non-vitrified pre-antral follicles after 0, 24, 48, 72, and 96 h of culture with or without pretreatment of ALA are shown in Figure 3. At the initial time of cultivation period, ROS levels of fresh pre-antral follicles (1.7 µM) were significantly lower than those of vitrified-warmed pre-antral follicles (2.3 µM, *P*<0.05). Maximum ROS levels in the fresh pre-antral follicles (4.5 µM) without pretreatment of ALA were observed at 96 h of culture (*P*<0.05). However, in the presence of ALA, these levels occurred at the initiation time and 24 h (1.5 and 1.9 µM, respectively) of culture and then remained unchanged up to 96 h (2.1 µM). ROS levels were significantly increased in vitrified-warmed pre-antral follicles in the absence of ALA during the culture period up to 96 h (*P*<0.05); however, there was not observed any significant differences between the ROS levels at the end (2.3 µM) and the beginning (2.2 µM) of the culture in the vitrified-warmed pre-antral follicles following treatment with ALA (*P*>0.05). The ROS amount in fresh pre-antral follicles and vitrified-warmed pre-antral follicles were significantly lower at 48, 72 and 96 h of culture in ALA-supplemented groups compared to respective groups without pretreatment of ALA (*P*<0.05).

**Table 3:** Rates of developmental competence of MII oocytes derived from cultured fresh pre-antral follicles and vitrified-warmed pre-antral follicles supplemented with or without alpha lipoic acid (ALA)

**Groups**	**ALA**	**No. of MII oocytes**	**Fertilized** **n** **(% ± SD)**	**Two cells** **n** **(% ± SD)**	**Morula** **n** **(% ± SD)**	**Blastocytes** **n** **(% ± SD)**
Fresh follicles	**-**	103	67^a^ (65.1 ± 2.9)	45^a^ (43.6 ± 2.1)	31^a^ (30.1 ± 3.1)	25^a^ (24.3 ± 0.9)
					
	**+**	97	81^b^ (83.6 ± 3.8)	54^b^ (55.6 ± 2.2)	47^b^ (48.6 ± 2.5)	41^b^ (42.3 ± 1.5)
						
Vitrified pre-antral follicle	**-**	54	29^c^ (53.6 ± 4.0)	19^c^ (35.1 ± 1.8)	11^c^ (20.3 ± 2.4)	9^c^ (16.7 ± 0.9)
					
	**+**	66	42^a^ (63.6 ± 4.2)	28^a^ (42.4 ± 2.8)	22^a^ (33.2 ± 3.2)	17^a^ (25.8 ± 1.3)

In all cases at least 4 experimental replicates were performed. Different superscripts in the same column reflect different levels of significant difference (*P*<0.05). MII, metaphase II oocyte

## DISCUSSION

The present study shows that ALA can improve developmental competence of vitrified-warmed isolated pre-antral follicle *via* reduction of ROS production and compensation of diminished TAC levels during cultivation period. 

Our results showed that the production of ROS had a tendency to be increased during cultivation period, while this situation was intensified in vitrified-warmed samples. This finding is somewhat in agreement with other investigations that showed with increasing duration of cultivation period, ROS levels was increased [6, 10]. It is known that mitochondrial electron transport chain is a major source of ROS production during normal conditions [18], and its ROS production increases under pathological conditions [19]. It seems that elevated production of ROS in the *in vitro* culture condition is an inevitable event, because in addition to endogenous sources of ROS originating from cellular metabolism, exogenous factors, such as visible light, oxygen concentration, handling etc. enhance its production, which leads to oxidative stress [7, 8, 20]. This result is in agreement with the results of this study and our previous study [6] that showed ROS production was increased in cultured pre-antral follicles up to 96 h. 

Effect of cryopreservation on ultra-structural properties of vitrified ovaries has been investigated previously [14, 21, 22]. In this continuum, evaluation of oocyte ultra-structure after vitrification and warming under transmission electron microscopy showed that the its low developmental competence was partially due to diffused and fragmented mitochondria [22]. Also, it was indicated that isolated oocytes from vitrified-warmed ovarian tissues revealed increased numbers of swollen mitochondria [14, 21]. Our explanation for different ROS production between vitrified and fresh samples is mitochondrial disorder that occurs throughout cryopreservation. 

Several defense mechanisms against oxidative stress are present within cells including non-enzymatic antioxidants and enzymatic defense mechanisms [20]. Impact of ALA as a potent antioxidant on the developmental competence of isolated pre-antral follicles in fresh samples was reported previously [6]; however, this is the first study to show the effects of ALA on the developmental competence of vitrified-warmed pre-antral follicles. We showed that ALA not only increased ROS level but also decreased TAC, which in turn improved the rates of survival and developmental competence of vitrified-warmed and fresh pre-antral follicles after long-term *in vitro* culture. Several pathways can be considered for the role of ALA in improving the development of vitrified-warmed and fresh pre-antral follicles. For example, efficiency of ALA as a unique antioxidant of lipoate/ dihydrolipoate system can imply ROS scavenging ability [6, 23]. In this sense, our finding showed that the ROS levels in fresh pre-antral follicles and vitrified-warmed follicles were significantly lower at 48, 72, and 96 h of culture with pretreatment of ALA than untreated groups. It has been also shown that ALA could suppress TNF-alpha-induced ROS generation [24] and 6-hydroxydopamine-induced ROS generation [11]. The other explanation for effects of ALA on improving *in vitro* maturation conditions may be related to inhibition of cell death, metal chelation, and antioxidant recycling [11]. ALA as a coenzyme for pyruvate dehydrogenase complex catalyzes the consumption of pyruvate, which is an essential source of energy during meiotic maturation [25]. 

The current study shows that TAC levels decrease significantly in both vitrified-warmed and fresh pre-antral follicles up to 96 h, whereas in the presence of ALA, there is no significant different among TAC levels of fresh pre-antral follicles between the beginning and end of the cultivation period. TAC levels of vitrified samples with pretreatment of ALA were also increased significantly after 24 h and remained persistent without any change up to 96 h later. In this regard, it has been demonstrated that plasma TAC levels increase after ALA administration [26]. This observation is in agreement with Akpinar *et al.* [27] finding that showed ALA through influence on the nerve growth factor induce expression of superoxide dismutase gene, which in turn increases superoxide dismutase. Also, it has been shown that ALA increases the catalase activity and glutathione peroxidase activity [27]. Catalase and glutathione peroxidase are two fundamental enzymes can be used as a biomarker of oxidative stress [28]. It has been also suggested that activation of glutathione peroxidase in follicular fluid is clearly associated with developmental competence of oocyte [29].

In conclusion, the results of the present study demonstrate that culture medium supplemented with ALA improves maturation of cultured vitrified-warmed pre-antral follicles through increasing follicular TAC level and decreasing ROS level. Future studies should therefore be conducted on the identification of metabolic role of ALA and their biochemical mechanisms, which are involved in follicular maturation, beside its antioxidative activity in assisted reproduction techniques.
